# Are They Really Stem Cells? Scrutinizing the Identity of Cells and the Quality of Reporting in the Use of Adipose Tissue-Derived Stem Cells

**DOI:** 10.1155/2016/2302430

**Published:** 2015-12-20

**Authors:** Ernesto Balolong, Soojung Lee, Judee Grace Nemeno, Jeong Ik Lee

**Affiliations:** ^1^Regenerative Medicine Laboratory, Center for Stem Cell Research, Department of Biomedical Science and Technology, Institute of Biomedical Science and Technology, Konkuk University, 120 Neungdong-ro, Gwangjin-gu, Seoul 143-701, Republic of Korea; ^2^Regeniks Co., Ltd., Seoul, Republic of Korea; ^3^Department of Veterinary Medicine, College of Veterinary Medicine, Konkuk University, Seoul 143-701, Republic of Korea

## Abstract

There is an increasing concern that the term adipose tissue-derived stem cell (ASC) is inappropriately used to refer to the adipose stromal vascular fraction (SVF). To evaluate the accuracy and quality of reporting, 116 manuscripts on the application of ASC in humans and animals were examined based on the 2013 published International Federation for Adipose Therapeutics and Science (IFATS)/ International Society for Cellular Therapy (ISCT) joint statement and in reference to current guidelines for clinical trials and preclinical studies. It is disconcerting that 4 among the 47 papers or 8.51% (CI 2.37–20.38) surveyed after publication of IFATS/ISCT statement reported using ASCs but in fact they used unexpanded cells. 28/47 or 59.57% (CI 44.27–73.63) explicitly reported that adherent cells were used, 35/47 or 74.47% (CI 59.65–86.06) identified expression of surface markers, and 25/47 or 53.19% (CI 14.72–30.65) verified the multilineage potential of the cells. While there are a number of papers examined in this survey that were not able to provide adequate information on the characteristics of ASCs used with some erroneously referring to the SVF as stem cells, there are more room for improvement in the quality of reporting in the application of ASCs in humans and animals.

## 1. Introduction

The use of adipose tissue-derived stem cells (ASC) has gained popularity as alternative to bone marrow derived stem cells or to human embryonic stem cells, particularly as the manner by which the source tissue is collected is less invasive compared to the former and does not have serious ethical issues compared to the latter. In view of pronouncements on the beneficial use of stem cells in the popular media [1], acknowledging the real therapeutic potential of stem cells is yet to be made as the scientific community is just starting to unravel their efficacy and safety [2].

There has been a major confusion though in the use of the term adipose tissue-derived stem cells, with some authors referring to the heterogeneous stromal vascular fraction (SVF) after centrifugation as stem cells. To provide guidance on this, the International Federation for Adipose Therapeutics and Science (IFATS) and International Society for Cellular Therapy (ISCT) in 2013 published a joint statement regarding the characteristics and differences of the two portions when derived from the adipose tissue with recommendations on how both should be ascertained [3]. A number of other guidelines and expert opinions also have been published in relation to the use of stem cells in clinical trials and the importance of reporting guidelines for preclinical studies [4–9].

Our objective is to determine the exact identity of the ASCs used in human patients and animal subjects as reported in published papers and the quality of reporting in reference to existing guidelines and expert recommendations.

## 2. Materials and Methods

The search engine Pubmed (http://www.pubmed.org/) was used to come up with the list of manuscripts and publications related to researches or clinical reports employing ASC in human patients and animal subjects from January 2011 to June 2015. For a report to be included in this survey, it must indicate adipose tissue-derived stem cells in either the title or the abstract. The keyword “adipose tissue-derived mesenchymal stem cells” combined with “clinical trial,” “therapy,” or “patient” was used to generate the list. We excluded from the list review articles and those reports that utilize ASC for* in vitro* experimentation only. Relevant articles were initially identified by the title and abstract and subsequently each paper was examined further by verifying whether indeed the use of ASC was reported and applied in either human patients or animal subjects. It is acknowledged that this search method was not exhaustive as there are manuscripts in journals that are not included in Pubmed.

We evaluated the papers in four key characteristics to ascertain the identity of cells used in reference to the IFATS and ISCT recommendations. These include (1) the use of expanded cells as treatment regimen, alone or in combination with other agents, (2) explicitly mentioning plastic adherent cells that were used, (3) phenotyping of surface markers, and (4) conducting multilineage differentiation of the expanded cells. We divided the papers further into two groups to examine the impact of the IFATS and ISCT statement on how authors would report the identities of the cells used. These were the before IFATS/ISCT statement group, comprised of papers published during the period 2011–2013, and the after IFATS/ISCT statement group, comprised of papers published on 2014-2015.

For papers utilizing ASC in human patients, the key areas considered were ethics (reported undergoing ethical review and approval for the study via an institutional review, reported obtaining an informed consent, and reported clinical trial registration), safety (processing the cells in Good Manufacturing Practice or GMP facility, testing for genomic stability, and contaminants prior to use), and cell characteristics (viability, number of cell passages, and number of cells administered). For papers utilizing ASC in animal subjects, the key areas considered were ethics (reporting of oversight and approval of the study via the Animal Care and Use Committee), study design (allocation to groups/randomization, calculation of sample size, and blinding), experimental animals (species, sex, age, and group size), and cell characteristics (viability, number of passages, and number of cells delivered).

## 3. Statistical Analysis

Data are presented as number, proportion, and percentages with binomial 95% confidence interval. Proportions were analyzed using Fisher Exact Test and post hoc analysis using the statistical software GraphPad Prism. The results are presented as percentages with 95% confidence intervals and a value of *P* < 0.01 was considered to be statistically significant.

## 4. Results

### 4.1. Overall Description of Selected Articles

The electronic search identified 623 articles. Based on the title, abstract, and description of the paper, a total of 149 papers were shortlisted after all the review articles, duplicates, and papers not relevant to the survey were removed. Only 116 papers were retrieved for evaluation after manuscripts not complying with our criteria were taken out further from the list [10–125]. All in all, 34 papers reported the use of ASC in human patients, 81 papers reported the use of ASC in animal subjects, and 1 paper reported the use of ASC in both human and animals. Based on the year of publication, 69 papers were grouped as before IFATS/ISCT statement group while 47 papers were determined to comprise the after statement group. 88.57% (31/35) of papers reporting application for human use were clinical trials while 11.43% (4/35) were case reports. 81.71% (67/82) of the papers involving animal use were human preclinical studies, 3.66% (3/82) were veterinary clinical trials, and 2.44% (2/82) were veterinary preclinical studies. 12.20% (10/82) were classified as basic research.  Table 1 summarizes the specific papers in this survey that were classified as human and veterinary clinical trial.

We observed in this survey varying sources of adipose tissue from where the lipoaspirates and cells were derived. Overall, 27.59% (32/116) had human patients as sources, 26.72% (31/116) utilized tissues from human donors, 27.59% (32/116) derived the tissues from animal subjects and 12.07% (14/116) from animal donors, 0.86% (1/116) reported utilizing human adipose tissue-derived stem cell from a commercial source, 0.86% (1/116) utilized tissues from human patients and human donors, another 0.86% (1/116) derived the tissues from human donor and animal subjects, and 2.59% (3/116) derived the tissues from a human donor and an animal donor, while the remaining 0.86% (1/116) did not indicate the source of the tissue. We also classified the papers according to the nature of cell transplantation whether it will be autologous, allogeneic, or xenogeneic. Most of the papers on human application reported autologous use at 80% (28/35), with 14.28% (5/35) being allogeneic; 2.86% (1/35) were not classified as no source was indicated while the last 2.86% (1/35) were both autologous and allogeneic. For animal studies, most are allogeneic at 42.68% (35/82) or xenogeneic at 40.24% (33/82). 12.20% (10/82) are autologous and 4.88% (4/82) are both allogeneic and xenogeneic. Majority of the reports at 85.34% (99/116) indicated benefits results with the use of ASC while 12.93% (15/116) indicated otherwise.

Various routes of administration of ASC in humans were observed in this survey as indicated in the papers depending on which site the cells are expected to settle. These include intra-arterial, intra-articular, intradermal, intramuscular, and intravenous injection at the submucosal, mucosal, and subcutaneous layer or transplanted with the scaffold material and tissue graft.  Table 2 summarizes the papers included in this survey that reported route of delivery of the ASCs.  Table 3 presents the carrier used to resuspend the stem cells before use or administration of the cells in humans as reported by specific papers included in this survey. These include the use of culture medium with human albumin, stabilized hyaluronic acid, lactated Ringer's solution, and normal saline with or without 1% human albumin. Majority of the papers did not indicate the specific medium used.

### 4.2. Impact of IFATS and ISCT Statement

**Figure 1:**
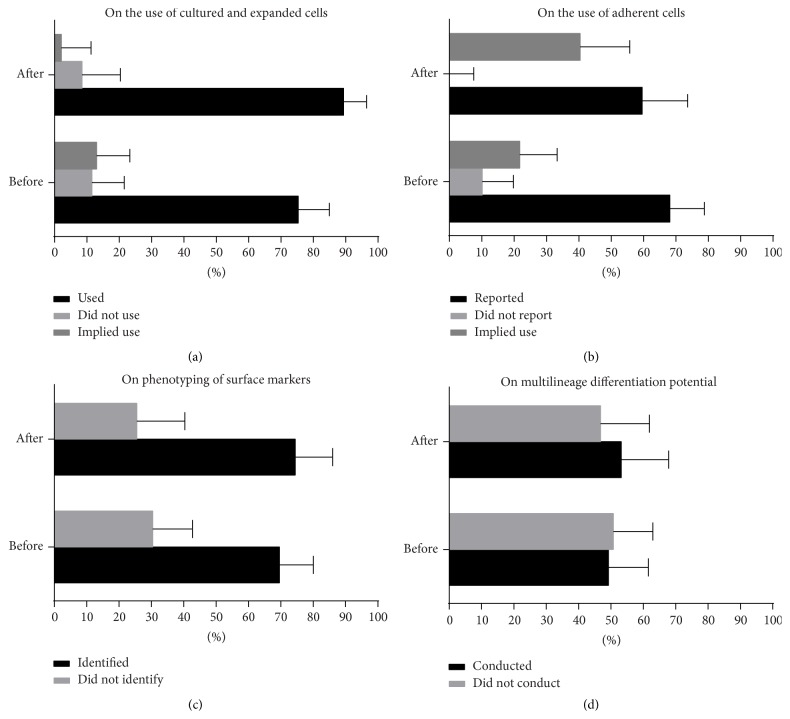
Impact of IFATS and ISCT on reporting of the characteristics of the ASC. Papers reporting on the characteristics of the cells transplanted or delivered to human patients or animal subjects were grouped and assessed before (*n* = 69) and after (*n* = 47) publication of the IFATS and ISCT statement.

**Figure 2:**
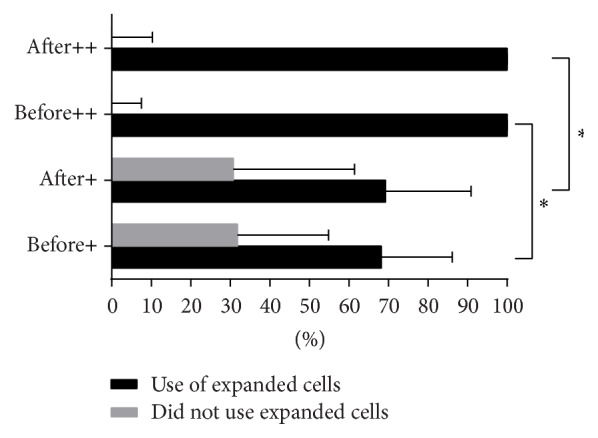
Disaggregated proportions of papers on human (+) and animal (++) application of ASCs that reported the use of expanded and unexpanded cells before and after IFATS/ISCT statement (before statement, human *n* = 22; after statement, human *n* = 13; before statement, animal *n* = 47; after statement, animal *n* = 34), percentage and 95% confidence interval, Fisher Exact Test *P* < 0.001 with post hoc analysis, and ^*∗*^significant difference *P* < 0.01.

Figure 1 illustrates the percentage of papers in reporting the primary characteristics of mesenchymal stem cells. There are still a number of papers which failed to conduct appropriate tests to verify the identity of the cells they use. Fisher Exact Test results showed significant difference in proportions (*P* < 0.05) in the before (*n* = 69) and after statement groups (*n* = 47) when reporting the use of adherent cells. There were no significance differences in the reporting of the surface markers and multilineage differentiation but there are a number of papers that failed to report such characteristics at the after statement group. 28/47 or 59.57% (CI 44.27–73.63) explicitly reported adherent cells (Figure 1(b)) were used and 35/47 or 74.47% (CI 59.65–86.06) identified expression of surface markers (Figure 1(c)). Meanwhile, 25/47 or 53.19% (CI 38.08–67.89) verified the differentiation potential of the cells (Figure 1(d)). Of these 25, 1 paper reported verifying only the osteogenic potential while 4 papers did the differentiation tests but nevertheless used unexpanded cells for the treatment groups. While there was no significant difference observed when comparing the before and after statement groups as far as the use of expanded cells is concerned (Figure 1(a)) disaggregating the data according to human (before statement, *n* = 22, after statement group *n* = 13) and animal (before statement group *n* = 47, after statement group *n* = 35) application showed significant difference (*P* < 0.001) and post hoc analysis indicates the difference when comparing papers with animal application to papers with human application of ASC (Figure 2). All of the 11 papers in this survey that did not use cultured and expanded cells but indicated they were using ASC instead of SVF were reports of application in humans. There is no significant difference between the human before statement group (7/22 or 31.82%, CI 13.87–54.87) and human after statement group (4/13 or 30.77%, CI 9.09–61.43).  Table 4 summarizes the frequency of papers that reported information on the characteristics of the cells used.

### 4.3. Quality of Reporting on ASC Use in Humans

**Figure 3:**
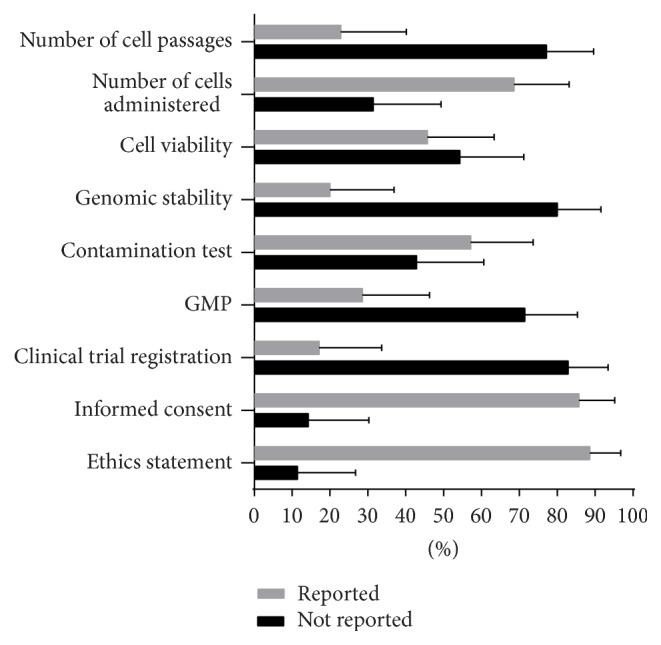
Quality of reporting papers on application of ASC in humans using selected parameters in ethics, safety, and cell characteristics (*n* = 35).

Figure 3 shows the percentages with 95% confidence interval of papers reporting on human application of ASC (*n* = 35) on selected parameters. There are 4/35 or 11.43% (CI 3.20–26.74) papers that did not indicate obtaining approval from an institutional review board. Of these 4, 3 reports were case studies while 1 paper was a preclinical study and, of the 4 case reports included in this survey, 1 paper reported having obtained an approval. 5/35 or 14.29% (CI 4.81–30.26) did not report having obtained informed consent from either patient or tissue donors. Of these 5, 2 papers were case reports while 3 papers were clinical studies. Only 10/35 or 28.57% (CI 14.64–46.30) reported processing the tissues and cells in GMP conditions. Of the 20/35 or 57.14% (CI 39.35–73.68) conducted tests for possible contamination before using the expanded cells, 1 paper reported testing for 5 different types of contaminants (viral, bacterial, fungal, mycoplasma, and endotoxin), 1 paper reported testing for 4 types, 6 papers reported testing for 2-3 types, and 11 papers reported testing for 1 type of contaminant. Of these 11, 10 papers tested for bacterial contamination, while 1 conducted endotoxin testing. More than half of these papers surveyed (19/35 or 54.28%, CI 36.35–71.89) did not report conducting cell viability while only 7/35 (20%, CI 8.44–36.94) checked the genomic stability of the cells. As to the number of cells delivered during transplantation 24/35 (68.57%, CI 50.71–83.15) reported actual cell numbers while 8/35 (22.86%, CI 10.42–40.14) indicated the number of passages at the time the expanded cells were used.  Table 5 summarizes the frequency of papers that reported on selected key areas and parameters of ASC use in humans.

### 4.4. Quality of Reporting on ASC Use in Animals

**Figure 4:**
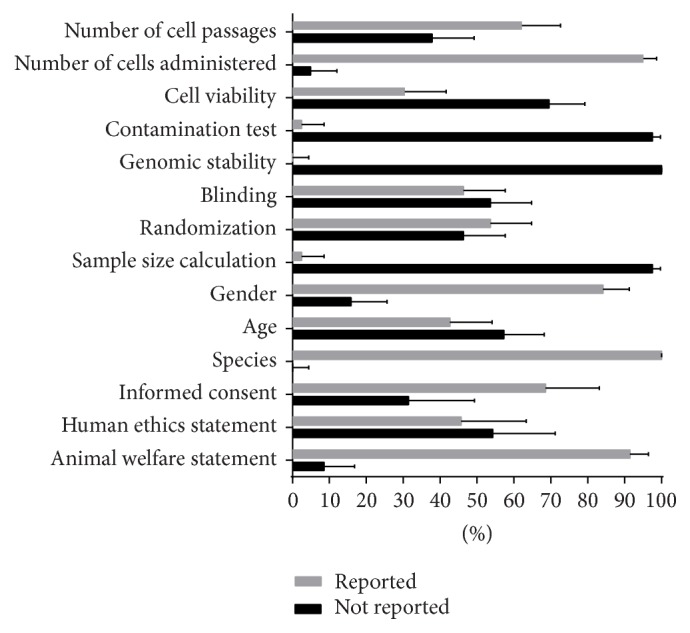
Quality of reporting papers on application of ASC in animals using selected parameters in ethics, safety, study design, and cell characteristics (*n* = 82).

Figure 4 presents percentages with confidence interval of papers reporting on animal application of ASC on selected parameters. Among the 82 papers reporting on animal application of ASC, there are 35 papers of those that utilized human adipose tissue as source for mesenchymal stem cells. Of these 35, 24 or 68.57% (CI 50.71–83.15) reported on obtaining informed consent from human donors while a lesser number of 16/35 (45.71%, CI 28.83–63.35) reported on having obtained approval from an institutional review board for the use of human samples. 7/82 or 8.54% (CI 3.5–16.80) did not report obtaining an approval from an animal welfare ethics committee. All papers reported the species used but 35/82 or 42.68% (CI 31.82–54.10) reported the age of their subjects while 69/82 or 84.15% (CI 74.42–91.28) reported whether male or female animals were used. 2/82 or 2.44% (CI 0.30–8.53) indicated how the sample size was calculated while 44/82 or 53.66% (CI 42.30–64.75) reported randomizing assignment of animals to treatment groups while 38/82 or 46.34% (CI 35.25–57.70) indicated that blinding techniques were done. No paper reported on conducting genomic stability tests for the stem cells used, 2/82 or 2.44% (CI 0.30–8.53) reported conducting testing for possible contaminants, 78/82 or 95.12% (CI 87.98–98.66) indicated the number of cells delivered, 25/82 or 30.49% (CI 20.80–41.64) reported checking the cell viability, and 51/82 or 62.20% (CI 50.81–72.68) reported the number of passages at the time the cells were used.  Table 6 summarizes the frequency of papers that reported on selected key areas and parameters of ASC use in animals.

## 5. Discussion

Transparency in reporting of research results is encouraged to hasten the standardization and reproducibility of the use of ASC for therapeutic purposes while highlighting the value of patient safety and product efficacy [126]. We assert further that accuracy in reporting the true identity of cells is very important especially that cell characteristics may vary according to source of tissue, species, site, physiological and disease condition, or age, may behave differently in a varying substrate or environment, and may provide differing outcomes when used for therapy [53, 67, 99, 104, 127, 128]. While the stem cell is a subset of the SVF it certainly is not one and, the same as the latter, contains more different types of cells other than the stem cells [3]. Expansion entails culturing plastic adherent cell after centrifugation to retrieve adequate cell population that is needed for transplantation purposes and we considered this as the primary parameter to gauge if indeed ASC was employed. Results in this survey show that despite guidance provided by IFATS and ISCT to ascertain the cells true identity still some authors claim that they used stem cells where in fact they did not. One paper goes to the extent of highlighting a rapid way of retrieving stem cells from the lipoaspirate in just 3 to 4 hours without any culturing done prior to injection. Although we cannot discount that using SVF may produce better or poorer results than using expanded mesenchymal stem cells, transparent reporting of the true identity of the cells should always be done. When we compared how reporting fared between papers on application of ASC in humans and in animals (Figure 2), reporting on human applications tend to misreport the identity of the cells used. How potentially serious the misreporting of stem cells instead of SVFs is shown by the 95% confidence interval for the proportion of these particular papers which was calculated to be at 2 to 20 percent.

Compliance to reporting standards and to expert's recommendation has been always a major concern even in the top-tier scientific journals [129]. The current survey showed that parameters on ethics, safety, study design, and cell viability in the application of stem cells to human patients as well as in animal subjects were not fully considered during reporting. Although the information we gathered from this survey is just based on the author's reports it may mean that these data were not reported as they were not done at all or were not reported but nevertheless were done. Poor quality in reporting will have an impact on our understanding of the real benefits of ASC and may seriously affect its potential to become a standard treatment. In addition, it may to some extent negatively affect, if not endanger, the patient when results from poor reporting are used as basis. For example, obscure quality and quantity of stem cells may lead to transplanting inadequate cells resulting in ineffective results, but if the cells are more than the required number, they may potentially circulate to unintended locations and form cell aggregates that could cause pulmonary emboli or infarctions [41, 112]. Patients are on the receiving end whenever medical institutions and companies processing stem cells for commercial purposes disregard proper protocols and standards to take advantage of the hype and exaggerated promotion of stem cells as a wonder treatment for intractable disease conditions. As to safeguarding the rights of recipients, it is not enough that an informed consent is obtained but an evaluation of the patient's understanding of the risks should also be secured [130]. The results of this survey showing misreports on the use of ASC may serve not only as a guide for readers to carefully examine published research results but also as a reminder for authors to prevent further misuse.

In the light of these survey results, we continually support the need for authors, reviewers, and editors to comply with the recommendations and guidelines. Such compliance not only should be reflected during reporting but must begin as early as the research planning or conceptualization stage until implementation. This survey is also a compelling evidence for the need to further disseminate the information and guidance provided by ISCT and IFATS or conduct further discussions in seminars or conferences. Researchers actively pointing out this similar oversight to editors will be helpful [131]. More research is required to fully understand the therapeutic potential, the effectiveness, and safety of ASC and a unified global effort to comply with the existing standards would definitely provide rapid and reliable results.

## 6. Conclusion

We determined in this survey that a substantial number of published reports were not able to provide information on the characteristics of ASCs used with some erroneously referring to the SVF as stem cells. In addition, survey results suggest that there is more room for improvement in the quality of reporting in the application of ASC in humans and animals in reference to existing guidelines and recommendations.

## Figures and Tables

**Table 1:** Papers included in this survey that reported on the use of ASC for human and veterinary clinical trials.

Classification	Source of adipose tissue for ASC	Type of transplantation	References
Human clinical trial	Human	Autologous	[10–39]
		Allogeneic	[37, 40–44]

Veterinary clinical trial	Animal	Autologous	[45, 46]
		Allogeneic	[47]

**Table 2:** Summary of papers that reported human application of ASC according to the route of delivery of stem cells.

Route of delivery	References
Intra-arterial	[37]
Intra-articular	[17, 42]
Intradermal	[11, 35]
Intramuscular	[10, 27]
Intravenous	[13–15, 41, 43, 44]
Injection at submucosal, mucosal, and tract wall	[12, 16, 28, 40]
Subcutaneous	[13, 14, 18, 26, 33]
Transplanted with scaffold material or tissue graft	[21, 26, 31, 38]

**Table 3:** Summary of papers that reported human application of ASC according to solution used to resuspend ASCs prior to delivery of cells.

Carrier used	References
Culture medium with human albumin	[40]
Hyaluronic acid	[33]
Lactated Ringer's solution	[32]
Normal saline	[11]
Saline with 1% human albumin	[14]

**Table 4:** Comparison on reporting of data on cell characteristics between papers grouped prior to and after publication of IFATS/ISCT statement on ASCs.

Cell characteristic	Description of data reported	Number of articles	
		Prestatement (*n* = 69)	Poststatement (*n* = 47)
Expanded cells	Use of expanded cells	52	42
	Did not use expanded cells	7	4
	Implied use	10	1

Adherent cells	Reported use Did not report Implied use	47 7 15	28 0 19

Phenotyping of surface markers	Phenotyping done No phenotyping	48 21	35 12

Differentiation potential	Conducted Did not conduct	34 35	25 22

**Table 5:** Summary of reporting on selected key areas and parameters in papers that reported the use of ASC in humans (*n* = 35).

Key area	Information	Number of articles	
		Yes	No
Ethics	Approval from review board Informed consent Clinical trial registration	31 30 6	4 5 29

Safety	Processing the cells in GMP Testing for genomic stability Screen contaminants	10 7 20	25 28 15

Cell characteristics	Viability Number of cells administered Number of cell passages	16 24 8	19 11 27

**Table 6:** Summary of reporting on selected key areas and parameters in papers that reported the use of ASC in animals (*n* = 82).

Key area	Information	Number of articles	
		Yes	No
Ethics	Approval from animal ethics committee	75	7
	Human ethics statement^*∗*^	16	19
	Informed consent from tissue donors^*∗*^	24	11

Animal characteristics	Species Age Gender	82 35 69	0 47 13

Study design	Random allocation to groups Calculation of sample size Blinding	44 2 38	38 80 44

Safety	Testing for genomic stability Screen contaminants Viability	0 2 25	82 80 57

Cell characteristics	Number of cells administered Number of cell passages	78 51	4 31

^*∗*^For papers that reported utilizing human tissue (*n* = 35).
